# 2-Nitroimidazole-Furanoside Derivatives for Hypoxia Imaging—Investigation of Nucleoside Transporter Interaction, ^18^F-Labeling and Preclinical PET Imaging

**DOI:** 10.3390/ph12010031

**Published:** 2019-02-15

**Authors:** Florian C. Maier, Anna Schweifer, Vijaya L. Damaraju, Carol E. Cass, Gregory D. Bowden, Walter Ehrlichmann, Manfred Kneilling, Bernd J. Pichler, Friedrich Hammerschmidt, Gerald Reischl

**Affiliations:** 1 Werner Siemens Imaging Center, Department of Preclinical Imaging and Radiopharmacy, Eberhard Karls University of Tübingen, Röntgenweg 13, 72076 Tübingen, Germany; florian.maier@med.uni-tuebingen.de (F.C.M.); gregory.bowden@med.uni-tuebingen.de (G.D.B.); walter.ehrlichmann@med.uni-tuebingen.de (W.E.); manfred.kneilling@med.uni-tuebingen.de (M.K.); bernd.pichler@med.uni-tuebingen.de (B.J.P.); 2 Faculty of Chemistry, Institute of Organic Chemistry, University of Vienna, Währingerstraße 38, A-1090 Vienna, Austria; anna.schweifer@univie.ac.at (A.S.); friedrich.hammerschmidt@univie.ac.at (F.H.); 3 Department of Oncology, University of Alberta, Edmonton, AB T6G 2R7, Canada; vd1@ualberta.ca (V.L.D.); ccass@ualberta.ca (C.E.C.); 4 Department of Dermatology, Eberhard Karls University of Tübingen, 72076 Tübingen, Germany

**Keywords:** tumor hypoxia, PET, small animal imaging, azomycin nucleosides, [^18^F]FMISO

## Abstract

The benefits of PET imaging of tumor hypoxia in patient management has been demonstrated in many examples and with various tracers over the last years. Although, the optimal hypoxia imaging agent has yet to be found, 2-nitroimidazole (azomycin) sugar derivatives—mimicking nucleosides—have proven their potential with [^18^F]FAZA ([^18^F]fluoro-azomycin-*α*-arabinoside) as a prominent representative in clinical use. Still, for all of these tracers, cellular uptake by passive diffusion is postulated with the disadvantage of slow kinetics and low tumor-to-background ratios. We recently evaluated [^18^F]fluoro-azomycin-*β*-deoxyriboside (*β*-[^18^F]FAZDR), with a structure more similar to nucleosides than [^18^F]FAZA and possible interaction with nucleoside transporters. For a deeper insight, we comparatively studied the interaction of FAZA, *β*-FAZA, *α*-FAZDR and *β*-FAZDR with nucleoside transporters (SLC29A1/2 and SLC28A1/2/3) in vitro, showing variable interactions of the compounds. The highest interactions being for *β*-FAZDR (IC_50_ 124 ± 33 µM for SLC28A3), but also for FAZA with the non-nucleosidic *α*-configuration, the interactions were remarkable (290 ± 44 µM {SLC28A1}; 640 ± 10 µM {SLC28A2}). An improved synthesis was developed for *β*-FAZA. For a PET study in tumor-bearing mice, *α*-[^18^F]FAZDR was synthesized (radiochemical yield: 15.9 ± 9.0% (*n* = 3), max. 10.3 GBq, molar activity > 50 GBq/µmol) and compared to *β*-[^18^F]FAZDR and [^18^F]FMISO, the hypoxia imaging gold standard. We observed highest tumor-to-muscle ratios (TMR) for *β*-[^18^F]FAZDR already at 1 h p.i. (2.52 ± 0.94, *n* = 4) in comparison to [^18^F]FMISO (1.37 ± 0.11, *n* = 5) and *α*-[^18^F]FAZDR (1.93 ± 0.39, *n* = 4), with possible mediation by the involvement of nucleoside transporters. After 3 h p.i., TMR were not significantly different for all 3 tracers (2.5–3.0). Highest clearance from tumor tissue was observed for *β*-[^18^F]FAZDR (56.6 ± 6.8%, 2 h p.i.), followed by *α*-[^18^F]FAZDR (34.2 ± 7.5%) and [^18^F]FMISO (11.8 ± 6.5%). In conclusion, both isomers of [^18^F]FAZDR showed their potential as PET hypoxia tracers. Differences in uptake behavior may be attributed to a potential variable involvement of transport mechanisms.

## 1. Introduction

Nowadays, the value of tumor hypoxia imaging for patient stratification, targeted and individualized therapy, and the monitoring thereof is undeniable [1,2,3,4,5,6]. The presence of tumor hypoxia is associated with increased tumor-aggressiveness, invasiveness, and therapy resistance [1,7,8]. While considerable effort has been made in the past decades, the full potential of hypoxia imaging, especially using specific tracers for positron emission tomography (PET) still has to be unraveled, as detection sensitivity, PET tracer target specificity, and the underlying molecular mechanisms by which these can be achieved are not entirely clear. Thus, there is still the need for optimization of hypoxia tracers; the mechanistic questions of the underlying principles of PET hypoxia tracer uptake and image contrast need to be answered as well. Of the numerous hypoxia tracer classes that have evolved, we investigated the class of 2-nitroimidazoles in the past, with a specific focus on ^18^F-labeled 1-(5′-deoxy-5′-fluoro-*α*-D-arabinofuranosyl)-2-nitroimidazole ([^18^F]FAZA) [9,10,11,12,13]. To date, the uptake mechanism of e.g. [^18^F]FAZA has not been identified in detail, yet it is assumed to be via passive diffusion, as an alpha-configurated nucleoside derivative should not be transported actively. It has been conceptualized in the past [13,14,15,16] that uptake and retention of 2-nitroimidazole-sugars in hypoxic tumor tissue can be altered by permutation of both, sugar moiety and stereochemistry at the anomeric carbon atom (2-nitroimidazole linked)—with the rationale to take advantage of transport mechanisms involving nucleoside transporters. Involvement of nucleoside transporters in the tissue uptake-process of 2-nitroimidazole-sugars was only investigated recently with *β*-allofuranose as C_6_-sugar [15]. Transport of nucleosides and nucleoside analog drugs is mediated by two unrelated protein families in humans, the SLC28 family of concentrative nucleoside transporters (hCNTs) and the SLC29 family of equilibrative nucleoside transporters (hENTs) [17]. The SLC28 family has three concentrative (hCNT1/2/3) members and the SLC29 family four equilibrative members (hENT1/2/3/4), respectively. The roles of human nucleoside transporters (hNTs) in transport of nucleosides and nucleoside drugs are summarized in recent reviews [18,19].

Here, we aimed at a systematic investigation of the interaction of selected 2-nitroimidazole-furanoses, resembling nucleoside analogs, with each of the five recombinant hNTs produced in a yeast model system (hENT1/2 and hCNT1/2/3). Transporters that may play a role in uptake of these nitroimidazoles into hypoxic tumor cells or normoxic control tissue like e.g. the muscle should be identified. The four compounds chosen for evaluation of their interaction with nucleoside transporters are found in Table 1, together with references to published data and the work list for the actual study. Besides the transporter interaction investigation, for *β*-FAZA, a novel synthesis route should be developed and for 1′-*α*-[2′,5′-dideoxy-5′-[^18^F]fluoro-D-ribofuranosyl]-2-nitroimidazole (*α*-[^18^F]FAZDR) ^18^F-radiolabeling should be established. In subsequent small animal PET imaging in a well-established tumor hypoxia model (colon carcinoma mouse model), *α*-[^18^F]FAZDR should be compared with [^18^F]FMISO, the gold standard in PET imaging of hypoxia [20,21] and *β*-[^18^F]FAZDR, of which positive PET imaging results have been recently obtained [13]. The *mouse* colon carcinoma model could be chosen, as mouse transporters exhibit close similarity to human nucleoside transporters [22]. With this comparative study, we sought to gain further insight into the importance and influence of the sugar moiety and configuration of 2-nitroimidazole at the anomeric carbon atom on tracer uptake and image contrast.

## 2. Results

### 2.1. Organic Chemistry

**Figure 1:**
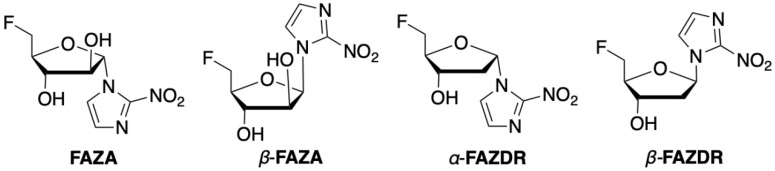
Chemical structures of the fluorinated 2-nitroimidazole C_5_-sugars FAZA, *β*-FAZA and *α*-, *β*-FAZDR, synthesized and used in the investigation of their interaction with human nucleoside transporters.

FAZA [23,24], *α*-FAZDR and *β*-FAZDR [13] were prepared by known procedures; for *β*-FAZA, a novel synthesis route was developed, although *β*-FAZA is a known compound [14]. Figure 1 gives the structures of the non-radioactive fluorinated 2-nitroimidazole sugars that were synthesized and evaluated for their interaction with human nucleoside transporters (see Section 2.3).

**Scheme 1:**
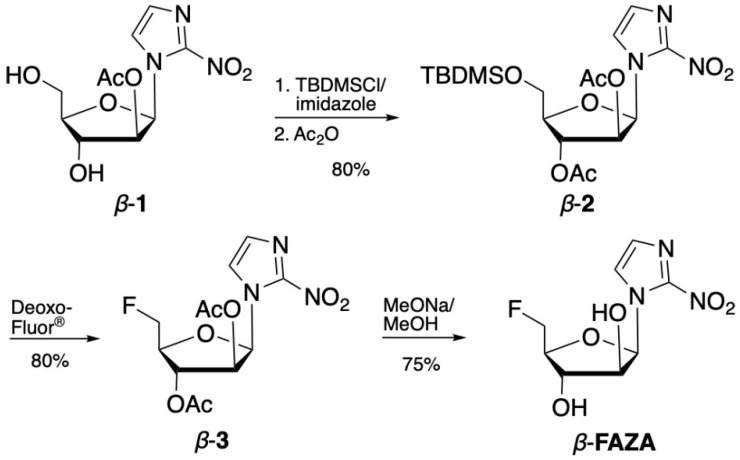
Three-step synthesis of the 5′-fluoro nucleoside analog *β*-FAZA, starting from 1-(2′-*O*-acetyl- *β*-D-arabinofuranosyl)-2-nitroimidazole (*β*-**1**; [25]). Reagents for and yields of the individual steps are given in the scheme.

An improved synthesis of the *β*-arabinose-derived nucleoside analog *β*-FAZA from the known starting material *β*-**1** (prepared as described by Kumar et al. [25]) was developed (Scheme 1). Selective silylation of the primary hydroxyl group at C-5′ with TBDMSCl/imidazole in pyridine, followed by acetylation, furnished crystalline and fully protected nucleoside *β*-**2** (80%). Bis(2-methoxyethyl)aminosulfur trifluoride [26] (Deoxo-Fluor^®^) converted it to 5′-fluoro nucleoside *β*-**3** in 80% yield. Deprotection with MeONa in MeOH gave the desired arabinose-derived nucleoside *β*-FAZA in 75%. *β*-FAZA was recently prepared by a different approach [14]. Here, we were able to improve the overall process compared to [14] by a reduction of synthetic steps (for initial reaction steps from the commercially available 1-*β*-D-(ribofuranosyl)-2-nitroimidazole, see [14,25]), overall reaction times, and an increase in yield to 48% for the three steps shown in Scheme 1, compared to 21% for the last three steps in [14]. The improvement was obviously due to the utilization of the commercial fluorination agent, Deoxo-Fluor^®^, in the penultimate step.

### 2.2. Radiochemistry

For a direct comparison with gold standard hypoxia PET imaging agent [^18^F]FMISO and the recently evaluated beta-isomer *β*-[^18^F]FAZDR [13] in a small-animal imaging study, alpha-ribofuranoside *α*-[^18^F]FAZDR was synthesized.

**Scheme 2:**
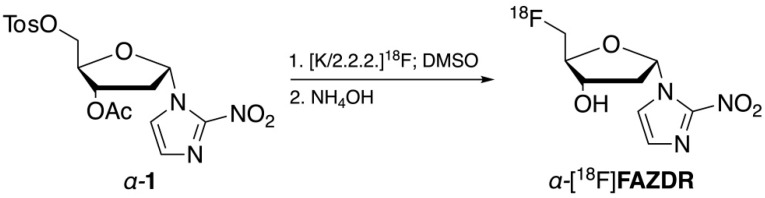
Radiosynthesis of *α*-[^18^F]FAZDR by nucleophilic substitution from the tosyl precursor *α*-**1** and subsequent hydrolysis with NH_4_OH.

Radiolabeling, hydrolysis, and purification were carried out similar to the synthesis of *β*-[^18^F]FAZDR (Scheme 2). Radiochemistry was performed on the same automated synthesis module (TRACERlab FX_F-N_, GE, non-cassette based) as for the other two PET tracers. Radiochemical yields were 15.9 ± 9.0% (*n* = 3) with higher variations compared to *β*-[^18^F]FAZDR (10.9 ± 2.4%, *n* = 4, uncorrected for decay [13]). Maximum yield obtained was 10.3 GBq at the end of the synthesis. As these amounts were sufficient for the planned mouse experiments and quality of the product was also satisfactory, the process and reaction parameters were not optimized further.

Radiochemical (RCP) and chemical purity (CP) were determined using HPLC and TLC. The latter method showed RCP of *α*-[^18^F]FAZDR of > 97%; in HPLC radiochromatograms, only the product peak was visible, corresponding to > 98% of RCP. With UV detection at 220 nm, only matrix peaks were visible (< 5 min retention time), with no other chemical impurities above the detection limit. The product solutions were always clear, colorless, and free of visible particles with a pH of 6.7 and a molar activity > 50 GBq/µmol.

### 2.3. Transporter Studies in Saccharomyces cerevisiae

The interaction of 2-nitroimidazole sugars with nucleoside transporters was determined by ability of the 2-nitroimidazole sugars to inhibit [^3^H]uridine uptake in yeast cells producing recombinant human nucleoside transporters and results expressed as IC_50_ values. Among the four compounds tested, only the *β*-sugar compounds inhibited hENT1 and hENT2 (SLC29A1/2), but at high concentrations. For FAZA and *α*-FAZDR, no interaction was detected. *β*-FAZDR inhibited hCNT1 (SLC28A1) and hCNT3 (SLC28A3) potently with IC_50_ values of 269 ± 4 µM and 124 ± 33 µM, respectively. FAZA inhibited hCNT1 with an IC_50_ value of 290 ± 44 µM and FAZA was the only compound to show considerable inhibitory activity against hCNT2 (SLC28A2) with an IC_50_ value of 640 ± 10 µM. Individual IC_50_ values for all compounds and investigated transporters are summarized in Table 2.

In summary, FAZA inhibited [^3^H]uridine transport by hCNT1 and hCNT2, in contrast to the commonly accepted theory of FAZA entering cells by passive diffusion. Although some of the compounds inhibited [^3^H]uridine uptake by nucleoside transporters, transporter interaction does not necessarily imply transport through the cell membrane. To confirm this uptake, experiments with the corresponding radioactive compounds needed to be undertaken, which was not possible here, due to their lack of availability. Keeping this in mind, it is nevertheless possible that our results challenge the current understanding of FAZA image contrast, as tumor-type dependent transporter expression might especially alter the early-phase uptake in tumor tissue and contribute to PET signal heterogeneity. This should be clarified in additional studies in the future. In contrast to FAZA, *β*-FAZA with the nucleosidic *β*-configuration showed weak interaction with the nucleoside transporters. Among the tested compounds, *β*-FAZDR showed best inhibition of uridine uptake by hCNT1 and hCNT3, while *α*-FAZDR showed no interaction with any transporter. Thus, both the deoxyribose sugar moiety and the *β*-configuration of the 2-nitroimidazole at the anomeric carbon atom are important molecular properties, if nucleoside transporter mediated uptake is envisaged in order to obtain higher TMR at early imaging time-points.

### 2.4. PET Hypoxia Imaging of CT26 Colon Carcinoma Bearing Mice

**Figure 2:**
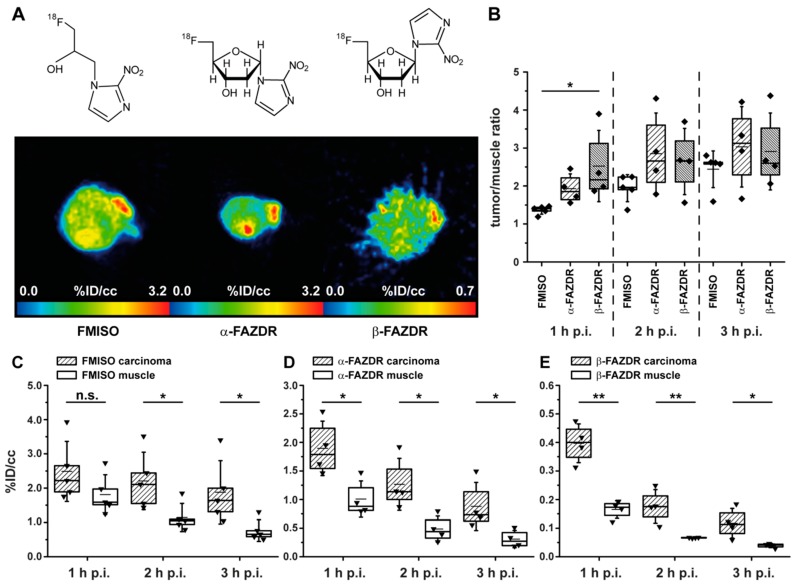
Exemplary structures and PET images of [^18^F]FMISO, *α*-[^18^F]FAZDR and *β*-[^18^F]FAZDR in carcinoma tissue 1 h p.i. (**A**), calculated tumor-to-muscle ratios (TMR) are shown in **B**. *β*-[^18^F]FAZDR (*n* = 4) displayed significantly higher TMR in comparison to [^18^F]FMISO (*n* = 5) 1 h p.i. (**B**), while *α*-[^18^F]FAZDR-TMR (*n* = 4) were not significantly different at any time-point. [^18^F]FMISO displayed significantly higher uptake in carcinomas vs. muscles only at 2 h and 3 h p.i. (**C**), while *α*-[^18^F]FAZDR and *β*-[^18^F]FAZDR at all measured time-points (1 h, 2 h and 3 h p.i.). * *p* < 0.05, ** *p* < 0.01 (**D**,**E**).

To build upon both, the obtained results from transporter studies in *Saccharomyces cerevisiae* and our recently published data on *β*-[^18^F]FAZDR PET imaging, we subsequently performed small animal hypoxia imaging with *α*-[^18^F]FAZDR and *β*-[^18^F]FAZDR in comparison with the clinical gold standard [^18^F]FMISO in CT26 colon carcinoma bearing BALB/c mice, a well-characterized tumor model for imaging hypoxia ([9,11,13], Figure 2A). Here, we were especially interested in the direct comparison of *α*-[^18^F]FAZDR and *β*-[^18^F]FAZDR, as the 2-nitroimidazole position at the anomeric carbon atom of 2-deoxyribofuranoside proved to be of importance for nucleoside transporter interaction.

First, we quantified [^18^F]FMISO uptake in hypoxic tumor tissue and in normoxic muscle tissue. While [^18^F]FMISO uptake was not significantly different at 1 h p.i. between tumor and muscle tissue (tumor: 2.49 ± 0.88 %ID/cc, muscle: 1.81 ± 0.58 %ID/cc), also reflected by the low TMR at 1 h p.i. (1.37 ± 0.11, *n* = 5), we obtained significantly higher uptake in tumors at 2 h and 3 h p.i. (Figure 2B,C). In direct comparison, we then investigated *α*-[^18^F]FAZDR (Figure 2A), where we expected hypoxia targeting in tumor tissue, but no major involvement of nucleoside transporters regarding tissue uptake. *α*-[^18^F]FAZDR-TMR at 1 h p.i. were higher (however, not significantly different) compared to TMR of [^18^F]FMISO at 1 h p.i. (*α*-[^18^F]FAZDR: 1.93 ± 0.39, *n* = 4; [^18^F]FMISO: 1.37 ± 0.11, *n* = 5; Figure 2B). Furthermore, *α*-[^18^F]FAZDR showed a significantly higher uptake in tumor tissue at 1 h, 2 h and at 3 h p.i. (1 h p.i. tumor: *1.90 ± 0.48 %ID/cc, muscle: *1.01 ± 0.32 %ID/cc; 3 h p.i. tumor: *0.88 ± 0.42 %ID/cc, muscle: *0.31 ± 0.15 %ID/cc; *n* = 4, * *p* < 0.05, Figure 2D). While the washout from both, tumor and muscle tissue of [^18^F]FMISO was limited, we observed an increased washout for *α*-[^18^F]FAZDR from both, tumor and muscle tissue (Figure 2D).

Next, we investigated *β*-[^18^F]FAZDR uptake and TMR. In good accordance with data recently published by us [13], we could again show a substantial washout of *β*-[^18^F]FAZDR from both, tumor and muscle tissue (Figure 2E). Furthermore, *β*-[^18^F]FAZDR was characterized by a significantly higher TMR in comparison to [^18^F]FMISO at 1 h p.i. (Figure 2B). From 0.40 ± 0.07 %ID/cc at 1 h p.i., the tumor uptake decreased to 0.18 ± 0.06 %ID/cc at 2 h p.i. and to 0.12 ± 0.05 %ID/cc at 3 h p.i., while normoxic muscle uptake decreased from the initially measured 0.17 ± 0.03 %ID/cc at 1 h p.i. to 0.07 ± 0.01 %ID/cc at 2 h p.i. and to 0.04 ± 0.01 %ID/cc at 3 h p.i. (*n* = 4, Figure 2E). This reproduced the results obtained in [13], also with regards to the TMR, which were 2.52 ± 0.94 at 1 h p.i., 2.65 ± 0.87 at 2 h p.i. and 2.91 ± 1.01 at 3 h p.i. (*n* = 4).

**Figure 3:**
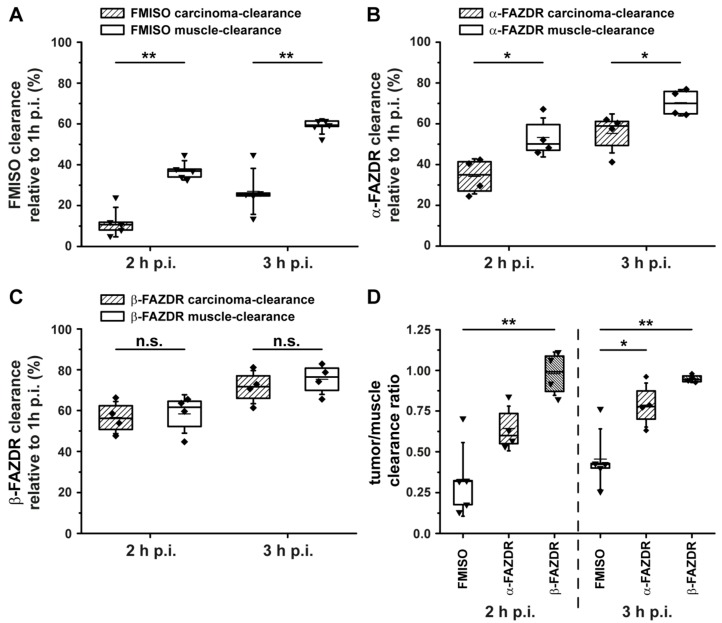
[^18^F]FMISO clearance was significantly different from carcinoma and muscle tissue (*n* = 5, **A**), however lower in comparison to clearance rates from both tissues for *α*-[^18^F]FAZDR (*n* = 4, **B**) and *β*-[^18^F]FAZDR (*n* = 4, **C**). Tumor/muscle-clearance ratios were significantly lower for [^18^F]FMISO in comparison to *β*-[^18^F]FAZDR at 2 h p.i. and to both, *α*-[^18^F]FAZDR and *β*-[^18^F]FAZDR at 3 h p.i. * *p* < 0.05, ** *p* < 0.01 (**D**).

To get a deeper understanding of this body of data, we also calculated tumor and muscle clearance for [^18^F]FMISO, *α*-[^18^F]FAZDR and *β*-[^18^F]FAZDR at 2 h and 3 h p.i. relative to 1 h p.i. Tumor clearance for [^18^F]FMISO was calculated to 11.8 ± 6.5% at 2 h p.i. (muscle clearance at 2 h p.i. 37.2 ± 4.3%) and to 26.9 ± 10.1% at 3 h p.i. (muscle clearance at 3 h p.i. 58.6 ± 3.3%, *n* = 5, Figure 3A); tumor clearance was significantly lower than muscle clearance for [^18^F]FMISO (p < 0.01). In direct comparison to [^18^F]FMISO, we observed higher tumor and muscle clearance rates for *α*-[^18^F]FAZDR (Figure 3B). Tumor clearance for *α*-[^18^F]FAZDR amounted to 34.2 ± 7.5% at 2 h p.i. (muscle clearance at 2 h p.i. 53.3 ± 8.3%) and to 55.3 ± 8.3% at 3 h p.i. (muscle clearance at 3 h p.i. 70.3 ± 5.5%, *n* = 4, Figure 3B) with significantly higher clearance rates from normoxic muscle tissue (*p* < 0.05). Finally, we quantified clearance rates for *β*-[^18^F]FAZDR, which turned out to be not significantly different between tumor and muscle tissue, reproducing our previously published results [13]. Overall, *β*-[^18^F]FAZDR tumor clearance was 56.6 ± 6.8% at 2 h p.i. (muscle clearance at 2 h p.i. 58.4 ± 8.2%) and 71.5 ± 7.0% at 3 h p.i. (muscle clearance at 3 h p.i. 75.3 ± 6.4%, *n* = 4, Figure 3C).

Next, we calculated clearance ratios for tumor relative to muscle tissue at 2 h and 3 h p.i. for a direct comparison between [^18^F]FMISO, *α*-[^18^F]FAZDR and *β*-[^18^F]FAZDR. Here, clearance ratios well below unity indicate higher clearance from muscle tissue, unity indicates equal clearance rates for both tumor and muscle tissue, and clearance ratios bigger than unity indicate higher clearance rates from tumor tissue. The latter case was not expected, as this would likely be the case for a rather hypoxia-unspecific tracer. Fitting the results of tracer-specific uptake behavior, TMR and clearance rates, [^18^F]FMISO showed the lowest clearance ratios (*, **0.45 ± 0.17, *n* = 5, 3 h p.i.), followed by *α*-[^18^F]FAZDR (*0.79 ± 0.12, *n* = 4, 3 h p.i.) and *β*-[^18^F]FAZDR with unitary clearance ratios (**0.95 ± 0.02, *n* = 4, 3 h p.i., * *p* < 0.05, ** *p* < 0.01, Figure 3D).

Conclusively, we observed the highest TMR for *β*-[^18^F]FAZDR at 1 h p.i. in comparison to [^18^F]FMISO and *α*-[^18^F]FAZDR, most probably mediated by the involvement of nucleoside transporters (Figure 2B). However, *β*-[^18^F]FAZDR was also characterized by the lowest net uptake in both tumor and muscle tissue in direct comparison with [^18^F]FMISO and *α*-[^18^F]FAZDR (Figure 2C–E), while [^18^F]FMISO- and *α*-[^18^F]FAZDR-uptake were comparable. Further confirming these findings, clearance rates from tumor tissue (and tumor-to-muscle clearance ratios) were lowest for [^18^F]FMISO, followed by *α*-[^18^F]FAZDR and *β*-[^18^F]FAZDR.

## 3. Discussion

While a lot of effort has been put in the development of hypoxia specific PET tracers (based on 2-nitroimidazole as hypoxia-selective moiety), and a wealth of studies indicate both hypoxia specificity and added value for patient stratification and treatment monitoring [1,2,3,4,5,6]; the exact image contrast generating mechanisms are still poorly understood. While the commonly accepted underlying theory assumes trapping of 2-nitroimidazole-based PET tracers in hypoxic tissue and washout from normoxic control tissue [12,23,27], a plethora of studies (both clinical and preclinical) have demonstrated that this is most probably not the only mechanism explaining the obtained image contrast for various 2-nitroimidazole-based PET tracers in diverse tumor-entities [9,13,28,29,30,31]. Especially, the fact that PET tracer kinetics of 2-nitroimidazole-based compounds can indicate both reversible or irreversible uptake in patients with the selfsame tumor type, e.g. head and neck cancer, challenges the concept of hypoxia-selective trapping [31]. This ambiguous behavior was also observed by others in both mouse and man [28,32,33]. Substantial tumor and muscle washout could recently be observed by us for both [^18^F]FAZA [9] and *β*-[^18^F]FAZDR [13]. While rapid washout from normoxic control tissue (in this case muscle tissue) was anticipated, we were again struck by the high washout rates from hypoxic tumor tissue, especially for *β*-[^18^F]FAZDR, and also, to a lesser extent, *α*-[^18^F]FAZDR. However, for both *α*-[^18^F]FAZDR and [^18^F]FMISO, the clearance ratios indicate lower tumor clearance, fitting the common theory of the underlying principle of a hypoxia-detecting PET tracer. But, although the unitary clearance ratios imply it, this does not conclude that *β*-[^18^F]FAZDR is not a valid hypoxia marker. In fact, we could recently prove the tumor hypoxia specificity of *β*-[^18^F]FAZDR [13]. Thus, one could conclude from this body of data that hypoxia tracer image contrast consists of three different components, which are theoretically influenced by the contribution of nucleoside transporters (detailed analysis of hypoxia tracer kinetic modeling complexity can be found in [30]): (i) Free tracer in tissue or in the fractional blood volume of target and reference tissue, (ii) non-specifically bound in target and reference tissue, and (iii) specifically bound or trapped in target tissue. Looking at all examined time-points (1 h, 2 h and 3 h p.i.), the in vivo behavior of the 2-nitroimidazole tracers we used in this study, ([^18^F]FMISO, *α*-[^18^F]FAZDR and *β*-[^18^F]FAZDR), could be explained by reversible kinetics. However, theoretically, if the sum of free and non-specifically bound tracer in target tissue is higher than the actual specific binding in target tissue, the observed clearance could be dominated by the washout of the non-specific or free tracer, while the specific fraction of the PET signal does not necessarily need to be reversible to generate the observed contrast; this could also be caused by a smaller fraction of irreversible binding. In addition, nucleoside transporters actively contribute to early and, thus also to late PET image contrast. While we cannot draw conclusions on the dominance of free, non-specific or specific fractions, we can conclude that nucleoside transporters definitely influence image contrast, especially early image contrast, here at 1 h p.i. Furthermore, the hypoxia specificity of *α*-[^18^F]FAZDR could be indirectly proven in this manuscript, as *α*-[^18^F]FAZDR-uptake perfectly resembled *β*-[^18^F]FAZDR-uptake in the same tumor, imaged on consecutive days (Figure 2A).

**Figure 4:**
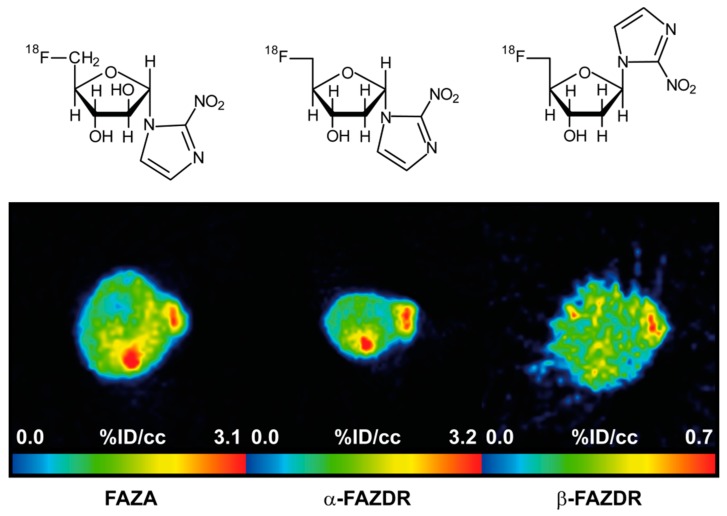
Exemplary scan of one CT26 colon carcinoma-bearing mouse with [^18^F]FAZA, *α*-[^18^F]FAZDR and *β*-[^18^F]FAZDR on three consecutive days, along with structures of used hypoxia tracers.

We additionally validated this finding by an exemplary scan of one CT26 colon carcinoma bearing mouse with [^18^F]FAZA, *α*-[^18^F]FAZDR and *β*-[^18^F]FAZDR on three consecutive days (Figure 4), displaying very similar uptake patterns. This can be concluded as the hypoxia selectivity of *β*-[^18^F]FAZDR, as recently shown by us [13], while [^18^F]FAZA hypoxia selectivity was shown by us and others in a plethora of publications [1,5,7,9,10,12,13,28,29,31]. In perfect concordance with the transporter data from this study, TMR at 1 h p.i. were highest for *β*-[^18^F]FAZDR, probably due to the involvement of nucleoside transporters.

Conclusively, the exact mechanism generating 2-nitroimidazole PET hypoxia tracer image contrast is still elusive, while we add knowledge on stereochemistry-dependent involvement of nucleoside transporters regarding both early and late PET signals of 2-nitroimidazole-sugars. For the first time, we could show an interaction of FAZA with nucleoside transporters, thus cellular FAZA-uptake may not solely be attributable to passive diffusion—although our results still need to be taken with caution, as interaction might not necessarily be related to active transport. These findings open up novel avenues for hypoxia imaging and might help clarifying the underlying principles of hypoxia imaging.

## 4. Materials and Methods

### 4.1. General

Acetonitrile for azeotropic drying before ^18^F-radiolabeling was from Merck (DNA synthesis grade, Darmstadt, Germany). Dimethylsulfoxide (DMSO, dried over molecular sieves) as solvent for labeling was used from Fluka (Germany). Kryptofix 2.2.2. was purchased from Merck.

FAZA [23,24], the precursor [13] for radiosynthesis of *α*-[^18^F]FAZDR (1-(3′-*O*-acetyl-2′-deoxy-5′-*O-p*-toluenesulfonyl-*α*-D-ribofuranosyl)-2-nitroimidazole (*α*-**1**)), *α*-FAZDR and *β*-FAZDR [13] were prepared by known procedures. All other chemicals and solvents (either Fluka or Merck) were of the highest purity available and used as received. Deuterated solvents were ordered from Eurisotop GmbH (Saarbrücken, Germany).

^1^H and ^13^C NMR spectra (*J*-modulated except for 2-nitroimidazole derivatives) were obtained from compounds dissolved in CDCl_3_, acetone-d_6_, and MeOH-d_4_ at 300 K using a Bruker AV 400 (^1^H: 400.13 MHz and ^13^C: 100.61 MHz) spectrometer. Chemical shifts were referenced to residual CHCl_3_ (δ_H_ = 7.24), CDCl_3_ (δ_C_ = 77.00), residual CHD_2_C(O)CD_3_ (δ_H_ = 2.05), CD_3_C(O)CD_3_ (δ_C_ = 30.50), residual CHD_2_OD (δ_H_ = 3.31) and CD_3_OD (δ_C_ = 49.00). IR spectra were measured of films on a silicon disk [34] or in ATR mode on a Bruker VERTEX 70 IR spectrometer. Optical rotations were measured at 20 °C on a PerkinElmer 351 polarimeter in a 1 dm cell. TLC was carried out on 0.25 mm thick precoated Merck plates; silica gel 60 F_254_. Flash (column) chromatography was performed with Merck silica gel 60 (230–400 mesh). Spots were visualized by UV and/or dipping the plate into a solution of (NH_4_)_6_Mo_7_O_24_^.^4H_2_O (23.0 g) and of Ce(SO_4_)_2_^.^4H_2_O (1.0 g) in 10% aqueous H_2_SO_4_ (500 mL), followed by heating with a heat gun. Melting points were determined on a Reichert Thermovar instrument and were uncorrected.

### 4.2. Synthesis of 1-(5′-Deoxy-5′-fluoro-β-D-arabinofuranosyl)-2-nitroimidazole (β-FAZA)

**1-(5′-*tert*.-Butyldimethylsilyl-2′,3′-di-*O*-acetyl-*β*-D-arabinofuranosyl)-2-nitroimidazole (*β*-2):** A solution of TBDMSCl (0.068 g, 0.45 mmol, 1.1 equiv.) in dry pyridine (1.43 mL) was added to a mixture of 1-(2′-*O*-acetyl-*β*-D-arabinofuranosyl)-2-nitroimidazole (*β***−1**; [25]) (0.118 g, 0.41 mmol) and imidazole (0.056 g, 0.82 mmol, 2 equiv.) at –20 °C under argon atmosphere. The mixture was stirred and allowed to warm to room temperature in the cooling bath within 18 h before Ac_2_O (0.27 mL) was added. After 1 h and addition of water (10 mL) and stirring for 15 min, the reaction mixture was extracted with EtOAc (3 × 7 mL). The combined organic layers were dried (MgSO_4_) and concentrated under reduced pressure. The residue was dried at 0.5 mbar and flash chromatographed (hexanes/EtOAc = 2:1, *R*_f_ = 0.56) to yield silylated and acetylated nucleoside *β***-2** (0.145 g, 80%) as colorless crystals; mp. 93-94 °C (*i*Pr_2_O, cooling from +50 °C to +4 °C); [*α*]_D_^20^ = +77.8 (*c* = 1.07, acetone). IR (Si): ν = 2931, 2858, 1754, 1542, 1482, 1370, 1239, 1098 cm^−1^; ^1^H NMR (400.13 MHz, CDCl_3_): δ = 7.79 (d, *J* = 1.1 Hz, 1H), 7.13 (d, *J* = 1.1 Hz, 1H), 6.79 (d, *J* = 5.3 Hz, 1H), 5.73 (dd, *J* = 5.3, 4.3 Hz, 1H), 5.36 (dd, *J* = 5.8, 4.3 Hz, 1H), 4.11 (td, *J* = 5.8, 3.4 Hz, 1H), 3.92 (AB part of ABX system, *J*_AB_ = 11.4 Hz, *J*_AX_ = *J*_BX_ = 3.4 Hz, 2H), 2.08 (s, 3H), 1.79 (s, 3H), 0.92 (s, 9 H), 0.11 (s, 3H), 0.106 (s, 3H) ppm; ^13^C NMR (100.61 MHz, CDCl_3_): δ = 169.6, 168.6, 144.4, 128.1, 123.2, 86.7, 82.3, 75.0, 73.9, 61.3, 25.8 (3C), 20.6, 20.0, 18.3, −5.5, −5.6 ppm. Analysis calcd for C_18_H_29_N_3_O_8_Si (443.53): C, 48.75%; H, 6.59%; N, 9.47%: Found: C, 48.73%, H, 6.49%; N, 9.42%.

**1-(2′,3′-Di-*O*-acetyl-5′-*O*-deoxy-5′-fluoro-*β*-D-arabinofuranosyl)-2-nitroimidazole (*β*-3):** A solution of 1-(5′-*tert*.-butyldimethylsilyl-2′,3′-di-*O*-acetyl-*β*-D-arabinofuranosyl)-2-nitroimidazole (*β***-2**) (0.117 g, 0.264 mmol) and Deoxo-Fluor^®^ (0.22 mL, 0.528 mmol, 2 equiv., 50% in toluene) in dry 1,2-dichloroethane (0.23 mL) was stirred and heated at 75–80 °C under argon for 2 h. The mixture was cooled at 0 °C, diluted with a saturated aqueous solution of NaHCO_3_ (10 mL) and extracted with EtOAc (3 × 10 mL). The combined organic layers were dried (Na_2_SO_4_) and concentrated under reduced pressure. The residue was flash chromatographed (hexanes/EtOAc = 2:1, *R*_f_ = 0.23) to give 5′-fluoro nucleoside *β*-**3** (0.070 g, 80%) as colorless needles; mp. 113–114 °C (CHCl_3_/*i*Pr_2_O, +50 °C to −27 °C) (lit.: 112–114 °C [14]); [α]_D_^20^ = +62.4 (*c* = 0.75, acetone). IR (Si): ν = 3163, 2992, 1758, 1542, 1483, 1369, 1232, 1055 cm^−1^. The NMR spectroscopic data are in agreement with those in the literature [14]. Analysis calculated for C_12_H_14_FN_3_O_7_ (331.26): C, 43.51%; H, 4.26%; N, 12.69. Found: C, 43.59%, H, 4.13%; N, 12.60%.

**1-(5′-Deoxy-5′-fluoro-*β*-D-arabinofuranosyl)-2-nitroimidazole (*β*-FAZA):** A solution of MeONa in MeOH (1.7 mL, 0.2 M) was added to a solution of protected fluoro arabino nucleoside *β*-**3** (0.061 g, 0.184 mmol) in dry MeOH (2.7 mL) under argon at –20 °C. After stirring for 45 min at that temperature, the solution was neutralized with AcOH and concentrated under reduced pressure. The residue was flash chromatographed (hexanes/EtOAc = 1:2, *R*_f_ = 0.34) to furnish fluoro nucleoside *β*-FAZA (0.034 g, 75%) as yellowish crystals; mp. 148 °C decomp. (MeOH/*i*Pr_2_O, cooling to −18 °C); [*α*]_D_^20^ = +142.2 (*c* = 0.69, MeOH). IR (ATR): ν = 3175, 1536, 1482, 1352, 1250, 1158, 1098, 1075, 1043, 1021 cm^−1^. The NMR spectroscopic data are in agreement with those in the literature [14]. Analysis calcd for C_8_H_10_FN_3_O_5_ (247.18): C, 38.87%; H, 4.08%; N, 17.00%. Found: C, 38.89%, H, 3.95%; N, 16.94%.

### 4.3. Radiosynthetic Procedures

[^18^F]FMISO was synthesized according to [35] and [^18^F]FAZA, *β*-[^18^F]FAZDR following [13].

For synthesis of *α*-[^18^F]FAZDR, a TRACERlab FX_F-N_ automated system (GE Healthcare, Münster, Germany) was used. [^18^F]Fluoride was produced at a PETtrace cyclotron (GE Healthcare, Uppsala, Sweden) in a niobium target body from [^18^O]water (Rotem, Israel) via the ^18^O(p,n)^18^F nuclear reaction and beam currents of 35 µA. The [^18^F]fluoride was trapped on a Sep-Pak Light Accell Plus QMA anion exchange cartridge (Waters, USA, preconditioning: 10 mL 1 N aqueous NaHCO_3_, 10 mL H_2_O, 5 mL acetonitrile, 10 mL air). Subsequently, radioactivity was eluted with a mixture of 900 µL of acetonitrile and 100 µL of water containing 3.5 mg (25 µmol) of potassium carbonate and 15 mg (40 µmol) of Kryptofix 2.2.2. The solvent was evaporated (vacuum ca. 12 mbar) at 70 °C for 7 min and then at 120 °C for another 7 min. Labeling was carried out after addition of 5 mg of precursor (*α*-**1**) in 1 mL of DMSO at 70 °C for 5 min. After cooling to ca. 40 °C, 1 mL of 0.2 N NH_4_OH was added for hydrolysis (10 min). The reaction mixture was neutralized using 0.5 mL of 0.2 N NaH_2_PO_4_ and injected onto the semi-preparative HPLC column for purification. A Luna C18(2) 250 × 10 mm, 5 µm (Phenomenex, Aschaffenburg, Germany) and a mixture of 8% ethanol in 10 mM Na_2_HPO_4_ (*v*/*v*) as eluent were used. The product was eluted with a retention time of 24 min (k’ = 9.8; flow rate 5 mL/min), detected by UV (220 nm) and a radio detector. Product volume was ca. 5–7 mL. Finally, the product was sterile filtered through a 0.22 µm filter (Millex-GS, Millipore, USA). Overall synthesis time was 65 min. Activity was determined and a sample taken for quality control.

Identity, radiochemical and chemical purity of *α*-[^18^F]FAZDR were determined using an analytical HPLC system with a Phenomenex Luna C18(2) column (250 × 4.6 mm, 5 µm); eluent was 5% acetonitrile in water (*v*/*v*) and flow rate 2 mL/min. HPLC eluate was monitored by radio- and UV detector (220 nm) in series. In this system, the product eluted with a retention time of 27 min (k’ = 26). Radio-TLC was performed on silica gel plates (POLYGRAM SIL G/UV_254_, 40 × 80 mm, Macherey&Nagel, Düren, Germany) with ethylacetate as eluent. For analysis, a phosphor imager (Cyclone Plus, PerkinElmer, Rodgau, Germany) was used, showing an R*_f_*-value between 0.57–0.64 for the product.

### 4.4. Transporter Studies in Saccharomyces cerevisiae

*Saccharomyces cerevisiae* yeast was separately transformed with plasmids (pYPhENT1, pYPhENT2, pYPhCNT1, pYPhCNT2, or pYPhCNT3) encoding hNTs (hENT1, hENT2, hCNT1, hCNT2, or hCNT3, respectively) as described elsewhere [36,37]. Uptake of 1 µmol/L [^3^H]uridine (Moravek Biochemicals) into yeast was measured as previously described [37,38] using a semi-automated cell harvester (Micro96 HARVESTER; Skatron Instruments, Tranby, Norway). Yeast was incubated at room temperature with 1 µmol/L [^3^H]uridine in yeast growth media (pH 7.4) in the presence or absence (uninhibited controls) of graded concentrations (0–3 mM) of test compounds. The following compounds were used for transporter experiments: FAZA, *β*-FAZA, *α*-FAZDR, *β*-FAZDR. Uridine self-inhibition was used to determine maximum inhibition of mediated transport. Concentration–effect curves were subjected to nonlinear regression analysis using Prism software (version 4.03; GraphPad Software Inc.) to obtain the concentration of test compound that inhibited uridine uptake by 50%, relative to that of untreated cells (IC_50_ values). Each IC_50_ value determination was conducted with nine concentrations and four replicates per concentration and experiments were repeated two to three times.

### 4.5. Cell Culture

CT26 mouse colon carcinoma cells were cultured as previously described [9], and mycoplasma infections were checked once a month. The cells were a kind gift of Prof. Dr. med. Ralph Mocicat (Institute of Molecular Immunology, German Research Center for Environmental Health, Munich, Germany).

### 4.6. Animals

The above-described CT26 mouse colon carcinoma cells were subcutaneously inoculated in the right shoulder of female BALB/c mice (1 × 10^6^ cells in phosphate-buffered saline, PBS). Subsequently, the carcinomas were allowed to grow for a period of 13 days, reaching approximately 0.3 cm³. PET imaging experiments were conducted within day 14-16 post tumor inoculation. All animal experiments were conducted according to guidelines for the use and care of laboratory research animals under the German Animal Protection Law, and approved by local authorities (Regierungspräsidium Tübingen).

### 4.7. PET Imaging

[^18^F]FMISO (11.8 ± 1.2 MBq, *n* = 5), *α*-[^18^F]FAZDR (12.6 ± 1.3 MBq, *n* = 4) and *β*-[^18^F]FAZDR (11.7 ± 0.9 MBq, *n* = 4) were intravenously injected (via the tail vein) in female CT26 colon carcinoma bearing BALB/c mice 14–16 days post tumor inoculation. One animal was scanned on three consecutive days with [^18^F]FAZA, *α*-[^18^F]FAZDR and *β*-[^18^F]FAZDR; all other animals were scanned with only one PET tracer (either [^18^F]FMISO, *α*-[^18^F]FAZDR or *β*-[^18^F]FAZDR). All experiments were done using medical air as carrier gas for isoflurane anesthesia (1.5% isoflurane during PET scans, flow rate 0.4 L/min), including tracer injections, with a specialized vaporizer (Vetland, Louisville, KY, USA). Subsequent to the tracer injections, the animals were allowed to freely move in their home cages for an uptake period of 55 min, thus, breathing room air. After the uptake period, static 10 min PET scans were acquired at 1 h post injection (p.i.), 2 h p.i. and 3 h p.i. PET scans were performed with a Siemens Inveon dedicated PET (DPET, Siemens Healthcare, Knoxville, TN, USA). PET scans were then reconstructed with a two-dimensional ordered subset expectation maximization algorithm (OSEM2D with 16 subsets and 4 iterations). Image zoom was set to 1 and matrix size to 128 × 128 yielding a final PET image spatial resolution of 0.79 × 0.79 × 0.80 mm³. PET data were manually corrected for radioactive decay of ^18^F.

PET image analysis was performed using the PMOD 3.2_base 64 image viewing tool (PMOD Technologies, Zürich, Switzerland). Standardized volumes-of-interest (VOI, spheres with a diameter of 2 mm) were placed over the maximal activity in the carcinoma tissue (hypoxic target tissue), while the VOI placement in the shoulder muscle tissue (contralateral to carcinomas, normoxic control tissue) was done according to studies previously performed by our group [11,13]. Finally, PET data were expressed as percent injected dose per cubic-centimeter (%ID/cc) or tumor-to-muscle ratio (TMR).

### 4.8. Statistical Analysis

All statistical data analysis was preceded by tests for homoscedasticity and normality of PET data. These tests were performed using the Brown-Forsythe and the Shapiro-Wilk test with the Origin 8.0 Pro Software Package (OriginLab, Northhampton, USA). Normally distributed data were analyzed for statistically significant differences with the two-sample Student’s *t*-test, done with the JMP 11.1.1 software package (SAS Institute GmbH, Böblingen, Germany). Multi-group comparisons were performed with p-values adjusted according to the number of groups (Tukey Kramer correction). Data are shown as the arithmetic mean ± one standard deviation (SD), unless otherwise mentioned; box plots contain all individual data points, the 25th, 50th and 75th percentile, as well as the arithmetic mean and one standard deviation (indicated by whiskers). For all tests, except for multi-group comparisons, p-values below 0.05 were considered as statistically relevant.

## Figures and Tables

**Table 1:** 2-Nitroimidazole arabinose (FAZA) or deoxyribose (FAZDR) *α*-, *β*-derivatives with references to published data or to be investigated in this study (*), regarding transporter interaction, ^18^F-radiolabeling and small animal PET imaging.

Compound	Transporter Interaction	^18^F-Radiolabeling	PET *In Vivo* Data (^18^F-Labeled; Small Animal Studies)
**FAZA**	*	[12]	[9,10,11,12,13]
***β*-FAZA**	*	[14]	[14]
***α*-FAZDR**	*	*	*
***β*-FAZDR**	*	[13]	[13] and *

**Table 2:** Inhibition of [^3^H]uridine transport by the 2-nitroimidazole sugar compounds in yeast cells producing each of the five recombinant hNTs in concentration-effect experiments, as described in Methods. IC_50_ values (calculated from dose-response curves) are presented as the mean ± SE for *n* = 2–3 experiments. (NI = no interaction).

Compound	hENT1	hENT2	hCNT1	hCNT2	hCNT3
	(SLC29A1)	(SLC29A2)	(SLC28A1)	(SLC28A2)	(SLC28A3)
			IC_50_ (µM)		
FAZA	NI	NI	290 ± 44	640 ± 10	1385 ± 100
*β*-FAZA	2070 ± 50	>3000	>3000	>3000	2309 ± 352
*β*-FAZDR	NI	>3000	269 ± 4	NI	124 ± 33
*α*-FAZDR	NI	NI	NI	NI	NI
